# Pertussis–SARS-CoV-2 Co-infection in Infants at Mohammed VI University Hospital, Marrakech, Morocco

**DOI:** 10.7759/cureus.103852

**Published:** 2026-02-18

**Authors:** Chaima Misab, Houssam Chait, Siham Karrati, Taoufik Ben Houmich, Asmae Lamrani Hanchi, Nabila Soraa

**Affiliations:** 1 Department of Microbiology, Faculty of Medicine and Pharmacy of Marrakech-Cadi Ayyad University, Mohammed VI University Hospital, Marrakech, MAR

**Keywords:** co-infection, infants, morocco, pertussis, sars-cov-2, syndromic pcr

## Abstract

Background: Viral-bacterial co-infections may worsen the clinical course of COVID-19; however, the clinical characteristics of pertussis co-infection with SARS-CoV-2 in infants remain poorly described.

Methods: We conducted a retrospective study (from January 2024 to September 2025) at Mohammed VI University Hospital in Marrakech. Infants younger than 24 months with clinical suspicion of pertussis underwent multiplex respiratory polymerase chain reaction (PCR) testing using the FilmArray Respiratory Panel 2.1 plus panel (BioMérieux, Marcy-l'Étoile, France). Co-infection was defined as the simultaneous detection of *Bordetella pertussis* and SARS-CoV-2 from the same nasopharyngeal specimen. Demographic, clinical, laboratory, and radiological data, as well as treatment and outcomes, were collected. Comparisons between groups were performed using Fisher’s exact test for categorical variables.

Results: Among 628 tested patients (including 419 children), 69 were diagnosed with pertussis. Nine infants (mean age: 0.08 years) had pertussis-SARS-CoV-2 co-infection. Typical pertussis features were present in all patients. Common findings included respiratory distress (6/9), oxygen desaturation requiring supplemental oxygen (4/9), wheezing (4/9), elevated C-reactive protein (CRP) (5/9), leukocytosis (9/9), lymphocytosis (7/9), including one case of marked lymphocytosis, and thrombocytosis (8/9), with platelet counts ≥ 600 G/L in three cases. Chest imaging revealed interstitial patterns (1/9), focal infiltrate (1/9), or hyperinflation (2/9). The median length of hospital stay was four days.

Conclusion: Pertussis-SARS-CoV-2 co-infection in early infancy is uncommon but clinically relevant. Syndromic PCR enabled prompt diagnosis, early initiation of macrolide therapy, and rapid implementation of isolation to limit transmission. Larger studies are needed to better define severity determinants and identify gaps in vaccine-preventable disease coverage.

## Introduction

*Bordetella pertussis* (*B. pertussis*) remains a vaccine-preventable respiratory infection that can be particularly severe in young infants because of narrow airways, immature immune responses, and the risk of apnea, cyanosis, leukemoid reactions, and pulmonary hypertension [1,2]. Despite widespread immunization, resurgent *B. pertussis* infection has been reported in multiple regions, driven by waning immunity, suboptimal vaccine coverage, diagnostic under-recognition, and possible pathogen adaptation [3-5]. In Morocco and comparable settings, gaps in coverage of the primary diphtheria-tetanus-pertussis series as well as delays in booster doses may leave infants susceptible to infection, often acquired from household contacts.

The COVID-19 pandemic introduced additional diagnostic and organizational challenges [6]. Clinical overlap between SARS-CoV-2 infection and pertussis including paroxysmal cough versus post-viral cough, apnea or desaturation in infants, feeding difficulties, and non-specific auscultatory findings may complicate clinical assessment and delay targeted therapy and appropriate isolation measures [6-8]. Although public health interventions during the pandemic temporarily reduced circulation of several respiratory pathogens, co-infections continued to occur and may be associated with increased healthcare needs in specific pediatric subgroups [7,9].

From a laboratory standpoint, syndromic multiplex polymerase chain reaction (PCR) panels have transformed urgent respiratory diagnostics by enabling the simultaneous detection of viral pathogens and key bacterial agents, including *B. pertussis*, from a single specimen with short turnaround times [5,10]. In early infancy (where lumbar puncture or extensive imaging may be debated), rapid microbiological clarification is particularly valuable to allow prompt initiation of macrolide therapy, appropriate isolation measures, and avoidance of cascades of low-yield investigations [5]. However, co-infection with *B. pertussis* and SARS-CoV-2 in infants has been only sparsely reported, and its clinical characteristics, short-term outcomes, and operational implications remain poorly defined [8,9].

The objective of this study is to describe the clinical characteristics, investigations, management, and outcomes of infants with confirmed *B. pertussis* and SARS-CoV-2 co-infection, a rare but clinically significant condition, to compare these features with infants infected only with *B. pertussis*, and to assess the practical value of syndromic multiplex PCR in guiding real-world pediatric care.

## Materials and methods

Design and setting

This was a retrospective observational study conducted at Mohammed VI University Hospital (Marrakech) within the departments of Microbiology and Pediatrics A, from January 2024 to September 2025.

Eligibility criteria

Eligibility criteria include infants younger than 24 months admitted with a clinical suspicion of pertussis. For the co-infected group, inclusion required the simultaneous detection of B. pertussis and SARS-CoV-2 by multiplex respiratory PCR on the same nasopharyngeal specimen. Infants with pertussis alone served as the comparison group.

Sampling and microbiological testing

Nasopharyngeal swabs were obtained at admission and analyzed using the FilmArray Respiratory Panel 2.1 plus (BioMérieux, Marcy-l'Étoile, France), detecting multiple common respiratory pathogens, including B. pertussis and SARS-CoV-2.

Data collection

Collected data included demographic characteristics, vaccination status, clinical symptoms, vitals and oxygen saturation (SpO₂), complete blood count (CBC) and C-reactive protein (CRP), imaging results, treatments (macrolides/other antibiotics, respiratory support), and clinical outcomes (intensive care unit, complications, and length of hospital stay).

Handling of missing data

For variables with missing values, the number of missing observations was documented. Cases with missing key outcome data were excluded from comparative analyses, while descriptive statistics included all available data. No imputation was performed.

Severity criteria

Severity criteria were defined a priori as the presence of at least one of the following: respiratory failure, need for ventilatory support, major lymphocytosis (>50 G/L), thrombocytosis (≥600 G/L), or organ dysfunction.

Statistics

Descriptive analysis was performed using counts and percentages for categorical variables, and means or medians for continuous variables, as appropriate. Comparisons between co-infected infants and those with only pertussis were performed using Fisher’s exact test for categorical variables, given the small sample size of the co-infected group and the presence of low expected cell counts. A p-value < 0.05 was considered statistically significant. Statistical analyses were conducted using Python (Python Software Foundation, Fredericksburg, VA, USA) with the SciPy statistical package.

## Results

During this period, 628 patients underwent high-panel respiratory PCR testing via nasopharyngeal swab, of whom 419 (67%) were children. The indication for respiratory PCR was validated by both a microbiologist and a clinician in all patients. Pertussis was confirmed in 69 patients with a mean age of six months, while co-infection with pertussis and SARS-CoV-2 was confirmed in only nine children with a mean age of one month. All patients presented with typical pertussis symptoms (cyanotic, emetogenic coughing fits with intervals between attacks), with specific clinical, radiological, and biological characteristics observed in each patient.

Clinical findings included fever in two patients, wheezing in four patients, crackles in two patients, snoring in three patients, respiratory distress and signs of respiratory effort in six patients, polypnea in three patients, and oxygen desaturation in four patients, requiring oxygen therapy. Deterioration in general condition was noted in two patients, and poor feeding in two patients. When compared with infants infected with pertussis alone, no statistically significant differences were observed for fever (23% vs. 19%, p = 0.6742), wheezing (45% vs. 36%, p = 0.7203), respiratory distress (67% vs. 35%, p = 0.1393), or need for oxygen therapy (45% vs. 20%, p = 0.1965). Core pertussis features were present in all infants in both groups (p = 1.00) (Table 1).

**Table 1:** Clinical characteristics of infants with pertussis with and without co-infection

Clinical characteristics	Co-infected infants (n)	Percentage	Pertussis-only infected infants (n)	Percentage	p-value
Fever	2	23%	11	19%	0.6742
Wheezing	4	45%	22	36%	0.7203
Respiratory distress	6	67%	21	35%	0.1393
Need for oxygen therapy	4	45%	12	20%	0.1965
Core pertussis features	9	100%	60	100%	1.0000

On radiological examination, an interstitial syndrome was observed in one patient, a focal pulmonary opacity in one patient, and chest distension in two patients. When compared with infants with only pertussis, no statistically significant differences were found regarding interstitial pattern (12% vs. 9%, p = 0.5824), focal consolidation (12% vs. 6%, p = 0.5140), or hyperinflation (23% vs. 14%, p = 0.6087) (Table 2).

**Table 2:** Imaging findings in co-infected versus pertussis-only infected infants

Imaging findings	Co-infected infants (n)	Percentage	Pertussis-only infected infants (n)	Percentage	p-value
Interstitial pattern	1	12%	5	9%	0.5824
Focal consolidation	1	12%	4	6%	0.5140
Hyperinflation	2	23%	8	14%	0.6087

Biological findings showed that the infectious assessment was positive, with elevated CRP in five patients and hyperleukocytosis in all patients. Hyperlymphocytosis was observed in seven patients, with major lymphocytosis in one case. Thrombocytosis was noted in eight patients, three of whom had platelet counts exceeding 600 G/L. Thrombocytopenia was identified in one patient, and monocytosis in five patients. Liver function tests were abnormal in one patient. Serum electrolytes and renal function tests showed no abnormalities, and additional microbiological investigations, including urine and sputum cytobacteriological examinations, were within normal limits. When compared with infants infected with pertussis alone, co-infected infants had significantly higher rates of leukocytosis (100% vs. 58%, p = 0.0214) and thrombocytosis (89% vs. 43%, p = 0.0134). No statistically significant differences were observed for elevated CRP levels (56% vs. 29%, p = 0.1318) or lymphocytosis (78% vs. 50%, p = 0.1610) (Table 3).

**Table 3:** Laboratory characteristics of co-infected versus pertussis-only infected infants CRP: C-reactive protein

Laboratory findings	Co-infected infants (n)	Percentage	Pertussis-only infected infants (n)	Percentage	p-value
Elevated CRP	5	56%	17	29%	0.1318
Leukocytosis	9	100%	35	58%	0.0214
Lymphocytosis	7	78%	30	50%	0.1610
Thrombocytosis	8	89%	26	43%	0.0134

Overall, while clinical and radiological characteristics did not differ significantly between co-infected infants and those with pertussis alone, co-infection with SARS-CoV-2 was associated with significantly higher rates of leukocytosis and thrombocytosis. Syndromic PCR testing confirmed dual detection of *B. pertussis* and SARS-CoV-2 in all nine patients, allowing rapid etiological attribution on the same day of sampling.

All patients were promptly treated with macrolides, and supplemental oxygen therapy was administered when clinically indicated in four of the nine cases. The median length of hospital stay was four days. No fatal outcomes were observed, and no patient required invasive mechanical ventilation.

In this retrospective series of nine infants with co-infection, classical features of pertussis predominated, frequently requiring oxygen supplementation, while hospital stays remained short. Syndromic PCR enabled rapid etiological confirmation prior to culture results, thereby supporting early targeted therapy and timely infection-control measures.

## Discussion

Previous studies have reported a high prevalence of respiratory co-infections among patients with *B. pertussis* (76.7%), predominantly affecting younger individuals, compared with cases of pertussis alone [10]. The underlying mechanisms responsible for this elevated rate of co-infection remain to be elucidated. One proposed explanation is that *B. pertussis*, in the context of an immature immune system, may create a permissive environment for secondary infections, thereby contributing to more severe clinical presentations. Notably, co-infections have been reported more frequently in patients with respiratory failure than in those without [5]. Several studies have shown that viral co-infection is common in respiratory infections associated with *B. pertussis*, with reported co-infection rates ranging from 47.2% to 90.9% [2,5,10].

Berry et al.'s descriptive study reported that the prevalence of *B. pertussis*-SARS-CoV-2 co-infection remains low but is associated with increased clinical severity. Demographic characteristics and disease severity differed between patients with and without co-infection. Overall, the findings suggest that pertussis-SARS-CoV-2 co-infection is more severe than pertussis alone, particularly with respect to seizures, hospitalization rates, and mortality. Despite the overall decline in reported pertussis cases during the pandemic, these data are clinically relevant, as they underscore that pertussis-SARS-CoV-2 co-infections do occur and are associated with a higher risk of complications and adverse outcomes. Recognizing this association is essential for healthcare providers to appropriately identify, prioritize, and manage high-risk populations [11].

In our study, nine infants met strict criteria for pertussis-SARS-CoV-2 co-infection on the same nasopharyngeal sample. The cohort displayed canonical pertussis features (paroxysmal cough with cyanosis and vomiting) with variable lower-respiratory involvement (respiratory distress, wheeze), inflammatory and hematologic signatures typical of infant pertussis (leukocytosis, lymphocytosis, and frequent thrombocytosis). Our findings align with reports that infant pertussis-even with a viral partner-tends to show lymphocytosis and thrombocytosis and may drive oxygen needs despite limited radiographic changes [10,12-14]. The absence of mortality in our small group is reassuring but should not downplay risk in early infancy, where apnea and pulmonary hypertension can complicate the course.

Our findings indicate that classical pertussis features predominated, while radiological findings were comparable to those observed in pertussis-only cases. However, co-infected infants showed significantly higher rates of leukocytosis and thrombocytosis compared to infants infected with only pertussis, suggesting an additive effect of viral infection on hematologic responses. Rapid syndromic PCR enabled timely diagnosis, early targeted therapy, and appropriate infection control measures. Although the sample size is limited due to the rarity of this co-infection, these findings provide valuable preliminary insights to guide early recognition and management, and further studies with larger cohorts are needed to confirm these observations and explore long-term outcomes.

Syndromic multiplex testing enabled rapid etiological identification on the first day of hospitalization, facilitating timely initiation of macrolide therapy and appropriate isolation measures, while avoiding unnecessary empiric broad-spectrum antibiotic use. In infants presenting with low-grade or overlapping clinical features, simultaneous pathogen detection reduced the risk of diagnostic anchoring on SARS-CoV-2 alone and supported targeted counseling regarding household transmission and vaccination status. These findings are consistent with the observed biological differences between groups, despite the absence of statistically significant differences in clinical and radiological presentations. The absence of statistically significant differences for certain clinical and radiological findings is likely related to the small number of co-infected infants included in our study, which limited statistical power and the ability to detect subtle but potentially clinically relevant differences.

The identification of pertussis in infants, including those within the pre- or peri-primary vaccination period, underscores the importance of addressing vaccination coverage gaps, promoting cocooning strategies among caregivers, and maintaining robust surveillance during and beyond COVID-19 waves. In addition, caregiver education should emphasize the need for early medical consultation in the presence of paroxysmal cough, apnea, or cyanosis.

The present study offers notable strengths, including laboratory-confirmed diagnoses; clearly defined inclusion criteria; comprehensive clinical, biological, and radiological data collection; and real-world data derived from a tertiary hospital setting. The use of syndromic multiplex PCR further enhanced rapid and accurate pathogen identification. Notable limitations include the small sample size, reflecting the rarity of *B. pertussis* and SARS-CoV-2 co-infection in infants, and the retrospective design. Despite these constraints, the findings provide valuable preliminary insights into the clinical and laboratory characteristics of this uncommon co-infection, which may inform early recognition and management in comparable clinical contexts.

## Conclusions

In early infancy, pertussis and SARS-CoV-2 co-infection is uncommon but clinically relevant. Syndromic PCR accelerates diagnosis, streamlines therapy and isolation, and supports stewardship, while culture remains indispensable. Strengthening vaccination coverage and rapid access to testing are complementary levers to reduce morbidity.
